# Public Perception of the COVID-19 Pandemic on Twitter: Sentiment Analysis and Topic Modeling Study

**DOI:** 10.2196/21978

**Published:** 2020-11-11

**Authors:** Sakun Boon-Itt, Yukolpat Skunkan

**Affiliations:** 1 Department of Operations Management, Center of Excellence in Operations and Information Management Thammasat Business School Thammasat University Bangkok Thailand; 2 Bangkok Christian Hospital Bangkok Thailand

**Keywords:** COVID-19, Twitter, social media, infoveillance, infodemiology, infodemic, data, health informatics, mining, perception, topic modeling

## Abstract

**Background:**

COVID-19 is a scientifically and medically novel disease that is not fully understood because it has yet to be consistently and deeply studied. Among the gaps in research on the COVID-19 outbreak, there is a lack of sufficient infoveillance data.

**Objective:**

The aim of this study was to increase understanding of public awareness of COVID-19 pandemic trends and uncover meaningful themes of concern posted by Twitter users in the English language during the pandemic.

**Methods:**

Data mining was conducted on Twitter to collect a total of 107,990 tweets related to COVID-19 between December 13 and March 9, 2020. The analyses included frequency of keywords, sentiment analysis, and topic modeling to identify and explore discussion topics over time. A natural language processing approach and the latent Dirichlet allocation algorithm were used to identify the most common tweet topics as well as to categorize clusters and identify themes based on the keyword analysis.

**Results:**

The results indicate three main aspects of public awareness and concern regarding the COVID-19 pandemic. First, the trend of the spread and symptoms of COVID-19 can be divided into three stages. Second, the results of the sentiment analysis showed that people have a negative outlook toward COVID-19. Third, based on topic modeling, the themes relating to COVID-19 and the outbreak were divided into three categories: the COVID-19 pandemic emergency, how to control COVID-19, and reports on COVID-19.

**Conclusions:**

Sentiment analysis and topic modeling can produce useful information about the trends in the discussion of the COVID-19 pandemic on social media as well as alternative perspectives to investigate the COVID-19 crisis, which has created considerable public awareness. This study shows that Twitter is a good communication channel for understanding both public concern and public awareness about COVID-19. These findings can help health departments communicate information to alleviate specific public concerns about the disease.

## Introduction

In the course of history, there have been many infectious disease outbreaks in the human population, causing both loss of life and damage to economies [1]. At the end of 2019, the World Health Organization (WHO) reported a cluster of cases of pneumonia in Wuhan. The cause of this pneumonia was later defined by the WHO as COVID-19. COVID-19 is a new infectious disease that is spread by respiratory droplets and contact and is generally infectious to human beings [2]. COVID-19 has had an unprecedented impact worldwide, with more than 10,000,000 confirmed cases and more than 500,000 reported deaths in more than 200 countries [3]. On January 30, 2020, the WHO reported that COVID-19 was a public health emergency of international concern [4].

Social media platforms can provide rich and useful information to predict and explain the characteristics and status of disease outbreaks [5]. Text mining can be used to extract health information from social media platforms such as Twitter [6]. Twitter data enable researchers to obtain large samples of user-generated content, thereby garnering insights to inform early response strategies. Social media data text mining has been used to track diseases and assess public awareness concerning health issues, enabling disease forecasting [7]. Text analysis of Twitter data is one of the most important areas of focus in medical informatics research [8].

COVID-19 is a scientifically and medically novel disease that is not fully understood, as it has yet to be consistently and deeply studied [9]. This study can be challenging because in the initial stage of an outbreak, most data are incomplete due to inadequate diagnostic and testing capabilities. Most of the data reported are epidemiological, such as data from medical units or scientific laboratories. The use of social media information to analyze syndromic surveillance, focusing on public health–related concerns using web-based information and content, is essential [10]. One important reason is that during an outbreak, social media plays a critical role, as these platforms reflect real-time public panic through comments. Twitter, one of these social media platforms, has often served as a communication modality during disease outbreaks [11]. Twitter provides rich information to increase public awareness and inform people about outbreak locations. This is very useful to provide insight regarding the issues related to infectious disease outbreaks.

Regarding COVID-19, there is a lack of social media data–based research studying the spread of the disease, the behavioral awareness of the public, and emergent conversations on COVID-19. Taking research published in 2020 as examples, Shen et al [12] studied mentions of symptoms and diseases on social media to predict COVID-19 case counts, and Huang et al [13] analyzed social media posts to study the characteristics of COVID-19 patients in China. However, both these studies focused primarily on China. Moreover, Park et al [14] addressed information transmission networks and news-sharing behaviors on Twitter regarding COVID-19 in Korea only. Abd-Alrazaq et al [15] conducted an infoveillance study on aspects of the COVID-19 pandemic, aiming to study the main topics of discussion related to the disease. Chen et al [16] presented basic statistics that tracked only Twitter activity responding and reacting to COVID-19–related events. Lwin et al [17] studied Twitter users’ public emotional responses to COVID-19, focusing on four basic emotions only. However, these previous studies did not use Twitter data to address conclusive themes, nor did they perform sentiment analysis during the timeline of COVID-19 in its initial stage. These missing data are important because themes and sentiment analysis can provide a wider overview of public awareness [18]. The evolution of sentiment analysis of Twitter data since the early stages of the COVID-19 pandemic has not yet been fully presented. Greater understanding and public awareness of the pandemic are still needed.

Building on previous research, this study posits that theme and sentiment analysis of posts on Twitter in the early stages of the COVID-19 pandemic can aid understanding of the emotions, beliefs, and thoughts of the general public. This is important to enable policy makers to increase situational awareness of COVID-19 and make suitable interventions during impending outbreaks. The objective is to answer two research questions: (1) What is the level of public awareness in terms of sentiments and emotions toward COVID-19? and (2) What are the emergent topical themes and discourses regarding COVID-19?

## Methods

### Data Collection

This study was conducted in two stages: (1) data collection using the Twitter streaming application programming interface (API) to collect COVID-19–related posts in the English language, and (2) data analysis to identify trends, keywords, and themes. The objective of this study was to answer questions relating to themes, public concerns, and sentiments regarding the COVID-19 pandemic through social media analytics. The data were collected from Twitter to build a database on the pattern of the COVID-19 epidemic. Data from Twitter are acceptable for research, being very rich and useful [19]. Twitter is a medium in which millions of people can express their views on any issue or topic. For example, during previous events such as natural disasters or disease outbreaks, people used Twitter to express their feelings [20,21]; this has been particularly true during the global epidemic of COVID-19. This study collected tweets using the Twitter streaming API, which is a Java application that can connect to a Twitter stream and store the raw data in a MySQL database. The Twitter streaming API enables nearly real-time access to the global stream of public tweets that match specified keywords. To access the API, it was necessary to be signed up on Twitter and log into the developer Twitter account. The next step was developing an application or API to provide the keys and tokens for using it in the programming environment.

The tweet database was created by specifying keywords and metadata such as language, source, data range, and location. This search used keywords and specific hashtags (#) such as *coronavirus*, *covid_19*, *2019-nCov*, and *covid-19* in the English language using the searchtweets tool [22]. The scope of the keywords and specific hashtags determines which tweets are delivered on the stream. This study employed a purposive sampling approach of tweets by active Twitter users between December 13, 2019, and March 9, 2020 (approximately covering the most recent days in the Twitter database). The main objective was to answer the research questions between the end of 2019 and the beginning of 2020; during this period, the outbreak started in China and then spread to Europe and America. This particular period is important to examine public concerns relating to the early COVID-19 outbreak.

The aim of our analysis of COVID-19 pandemic trends in English-language tweets was not to determine the volume of daily tweets. Our purpose was to measure the intensity of Twitter activities relating to COVID-19 with the stipulated keywords based on tweets that received retweets, representing conversation and content sharing about COVID-19 on Twitter [23]. This approach is effective because it will filter out low activity and outliers that are not useful. A sample of the top 1000 tweets with retweets as a proxy for Twitter activity was taken. Because the data were sampled on a daily basis over a period of 3 months, the granularity was sufficient to measure the changes in activity from day to day. This sampling process was repeated to collect a total of 107,990 tweets during this time period.

### Data Analysis

This study employed three data preparation steps: sampling, data collection, and preprocessing of raw data. To start data processing, the tweet texts were subjected to a series of functions to remove URLs, emojis, special characters, retweets, hash symbols, and hyperlinks pointing to websites; this process also enabled us, as much as possible, to exclude mentions of related diseases that would contaminate the results. Stop words in English (eg, *for*, *the*, *is*) were also removed, as were words such as *corona* or *virus* that may relate to other topics [24]. In addition, the tweet texts were converted to lower case, and words were changed to their root forms (eg, *viruses* to *virus*). The tweets were then converted into a corpus (text mining structure); we also created a document-term matrix and calculated the term frequency–inverse document frequency (TF-IDF), which is a numerical statistic used to reflect the importance of a word in a corpus. To obtain the output of the scenario, the tweet data were analyzed; to extract the tweet data, the Twitter API was used.

The data analysis not only focused on the overall picture of COVID-19 but was also scoped down based on keywords and specific hashtags such as *symptoms*, *outbreak*, and *pandemic*. The data analysis was conducted using Python software and RStudio. As mentioned earlier, three types of analysis were used to answer both research questions. First, the data analysis focused on the frequencies of single words (unigram) in the corpus of the text mining structure and visualized these frequencies through word clouds to display the most common topics. Content analysis can be used to analyze words or messages to show that events occurred after an incident or to study symptoms using word frequency counts, a widely used method in content analysis, as the rules for determining themes. The analysis also included time series using “retweet_count” and “favorite_count” as proxies of the intensity of social media activity on Twitter relating to COVID-19 to observe trends and timelines.

Second, sentiment analysis, a natural language processing (NLP) approach, was used to categorize the sentiments appearing in Twitter messages [25]. This approach involved analyzing the keywords appearing in the search topics and exploring the sentiments expressed in each topic related to COVID-19, including word frequency statistics and word clouds. For a more detailed analysis of the tweets, the emotional quotients associated with the tweets were analyzed. Sentiment analysis using the National Research Council (NRC) sentiment lexicon enabled us to examine the expression of 10 terms related to basic emotions: *anger*, *anticipation*, *disgust*, *fear*, *joy*, *negative*, *positive*, *sadness*, *surprise*, and *trust* [26,27]. The terms *positive* and *negative* were removed because they are classifications and do not indicate positive or negative emotions; also, emotions (eg, fear or joy) are indicated by the NRC sentiment lexicon. As a result, a total of eight emotions were evaluated in this analysis. Among the eight emotions, trust and joy were considered to be positive emotions, while anger, sadness, fear, and disgust were considered to be negative emotions. Surprise and anticipation could be either positive or negative depending on the context.

Finally, topic modeling based on unsupervised machine learning analysis was used to identify the most common topics in the tweets as well as to categorize clusters and find themes based on the keyword analysis. To perform topic modeling, the latent Dirichlet allocation (LDA) algorithm was applied. LDA is an unsupervised document classification method that is similar to clustering on numeric data; it can be used to find natural groups of items even when it is not certain what is being searched. The LDA algorithm is a particularly popular method for fitting a topic model. It treats each document as a mixture of topics and each topic as a mixture of words. In this process, tweeted messages can overlap each other in terms of content rather than be separated into different groups. In this study, we used the “tidy()” method, which is included in the broom package [28]. The “tidytext” package provides a method to extract the per-topic-per-word probabilities, called beta, from the model. To obtain the optimum number of topics, the main target was to compute the topic coherence for different numbers of topics and choose the model that gives the highest topic coherence. Coherence gives the probabilistic coherence of each topic. The coherence score is a score that indicates whether the words in the same topic make sense when they are extracted by those topics. The higher the score for a specific number k, the more closely related the words. Word clouds were used to represent topics that were classified based on the 10 most common keywords in each group.

## Results

### Twitter Trends During the COVID-19 Pandemic

**Figure 1:**
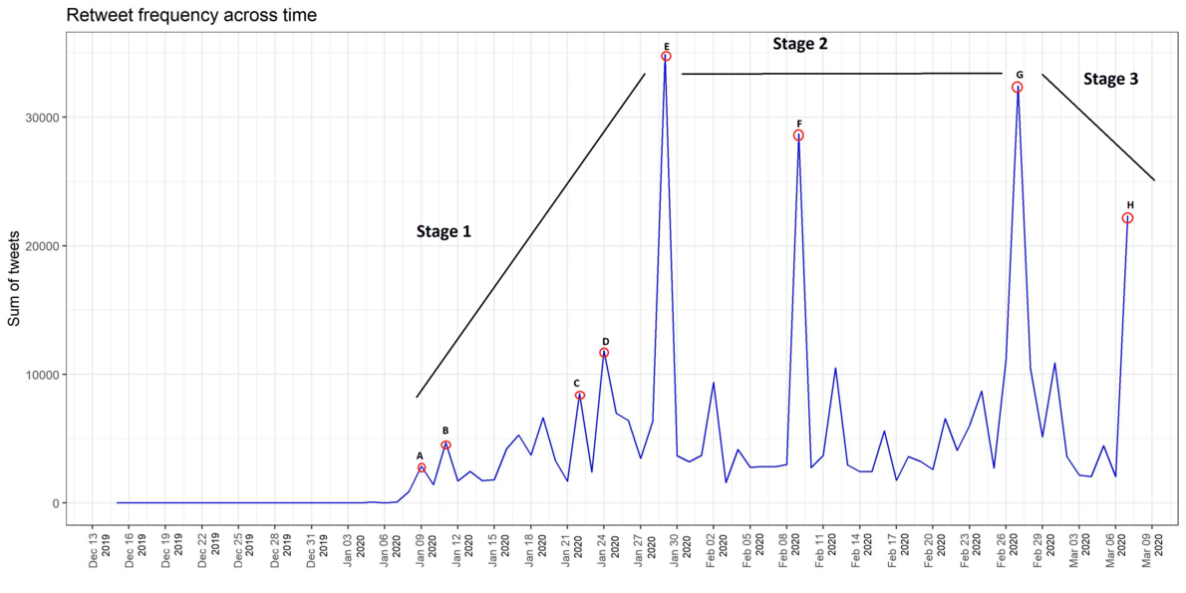
Frequency of retweets regarding the COVID-19 pandemic from December 13, 2009, to March 9, 2020. (A) A novel coronavirus was isolated; (B) the first fatal case was reported; (C) the first case of COVID-19 in the United States was confirmed; (D) 835 cases were reported in China; (E) the World Health Organization (WHO) declared a public health emergency of international concern; (F) the WHO announced the name “COVID-19”; (G) infections spiked in Italy and the rest of Europe; (H) the number of COVID-19 cases surpassed 100,000.

Figure 1 illustrates the retweet frequency of COVID_19–related tweets, showing that the trend line increased from January 7-9 to the first peak (Points A and B). The highest intensity peaks appeared from January 28-29 (Point E), with a second peak from February 9-11 (Point F) and a third peak from February 27-28 (Point G). The fourth peak appeared on March 6 (Point H). These results indicate public awareness, representing the intensity of conversation activities about COVID-19 on Twitter in the first period from January to its peak at the end of the month. This suggests the existence of an incubation period or early stage (Stage 1) when firsthand data about the severity of the emerging COVID-19 outbreak, including evidence of human-to-human transmission, started to appear. Data compilation of words related to symptoms of COVID-19 infection at the prodromal phase, including *fever*, *dry cough*, and *malaise*, was nonspecific. During the period from January 21-24 (Points C and D), the first confirmed American case of COVID-19 was declared in Seattle, and infection of health care workers was occurring. The spread had become more severe and general by the end of January, when the United States declared a public health emergency (Point E). In that period of time, the Tweet message intensity reached its first major peak.

Thereafter, the worldwide epidemic period began (Stage 2) during which the disease spread globally, negatively impacting health and economic activity. It is now known that during the epidemic period, the outbreak spread from China to other regions and countries, including Hong Kong, Taiwan, and Macau, as well as to East Asian countries such as Japan and South Korea. During this time, there was discussion on Twitter about the fatality rate, which was as high as 3%, until the panic or peak period at the end of January. The second major peak (Point F) occurred from February 7-9, when WHO officials announced that they had identified a new virus called 2019-nCoV (COVID-19), causing intense activity on Twitter. On February 27, when the third major peak began, COVID-19 reached Europe, and Italy saw a spike in the number of infections, which jumped to 650. It can be said that these two events indicated a worldwide pandemic, as COVID-19 spread from Asia to America and Europe after the first European outbreak in Italy. Later, policies such as social distancing and lockdowns were put in place. This was a stable stage (Stage 3) from the perspective of public awareness. Twitter intensity peaked again on March 6 (Point H), when the number of persons affected by COVID-19 surpassed 100,000. COVID-19 continued to spread even as the WHO urged countries to exert more effort to stop the spread of the disease [29].

**Figure 2:**
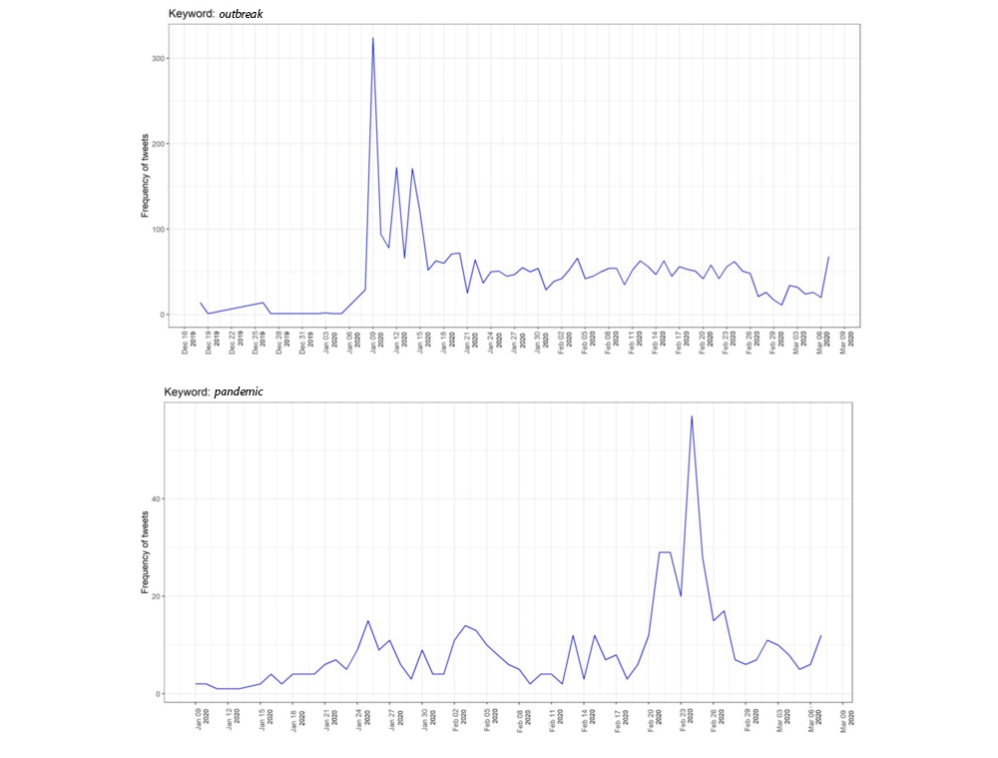
Frequencies of the keywords *outbreak* and *pandemic* on Twitter.

Figure 2 shows the Twitter intensities for two related but distinct keywords: *outbreak* and *pandemic*. The trend line for the keyword *outbreak* peaked between January 9 and 11. As the number of infections increased, Chinese officials stated that they had identified a new virus in the coronavirus family. It was initially named 2019-nCoV, which was later updated to COVID-19. This was the beginning of the outbreak of COVID-19, represented by high numbers of tweets including the word *outbreak*. Before COVID-19 reached the pandemic level, the trend line for the keyword *pandemic* peaked on February 24 as the virus spread from Asia to other continents. During that time, the WHO announced COVID-19 to be a worldwide epidemic that affected different countries in different ways. The word *pandemic* thus accurately described the situation.

### Twitter Trend Lines of COVID-19 Symptoms

**Figure 3:**
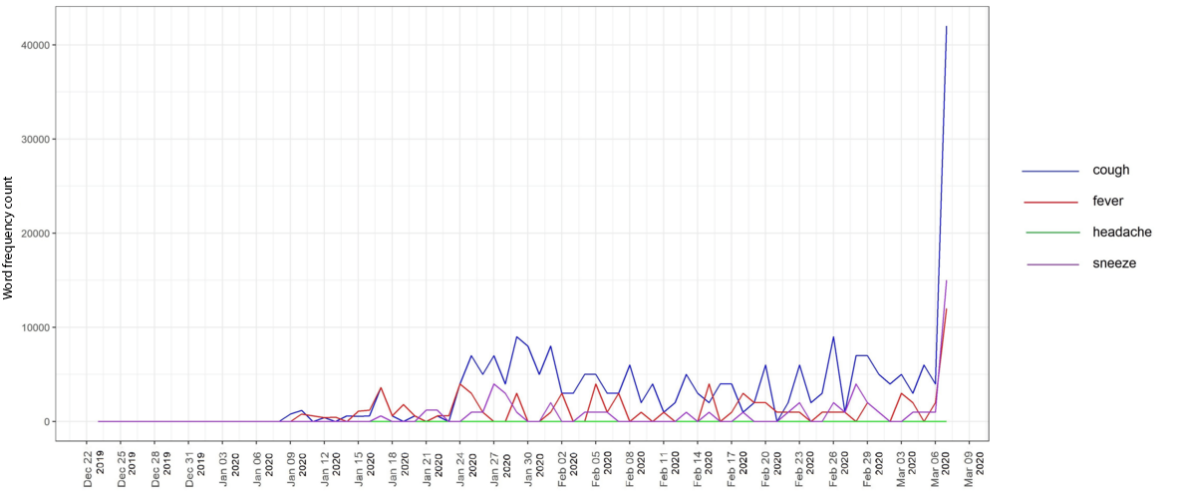
Trend lines indicating the word frequencies of four key COVID-19 symptoms on Twitter.

Figure 3 shows trend lines indicating the word frequency counts for the key symptoms of COVID-19 on Twitter, which may reflect users’ views and concerns about COVID-19 symptoms. The two key symptoms of COVID-19 are cough and fever [30]; common symptoms also include headaches and sneezing. Other symptoms (eg, body pain, runny nose, skin rash, frequent urination) are not shown in Figure 3 because only the four key symptoms were used to plot the graph. The word *pneumonia* was removed because this condition describes inflammation of the tissue in one or both lungs; we wished to look at other related symptoms and rank their daily Twitter data mentions to indicate public awareness and trends of COVID-19 symptoms.

Figure 3 presents the timelines of tweets mentioning symptoms of COVID-19. The analysis extracted messages mentioning at least one of the symptoms in the list. Before January 24, fever was mentioned most frequently, followed by coughing and sneezing. Headache had the lowest word frequency count. After January 24, coughing became a clear symptom and showed the most mentions, followed by fever and sneezing; meanwhile, headache was rarely mentioned, with no change in the trend as time passed. This suggests that fever is an early-stage symptom, leading to coughing, and headache may be a later symptom, with the fewest mentions. March 6 is an interesting date, as it shows the peak frequency of mentions of coughing and fever during the pandemic period examined in our study.

### Frequency of Keywords Related to COVID-19

**Figure 4:**
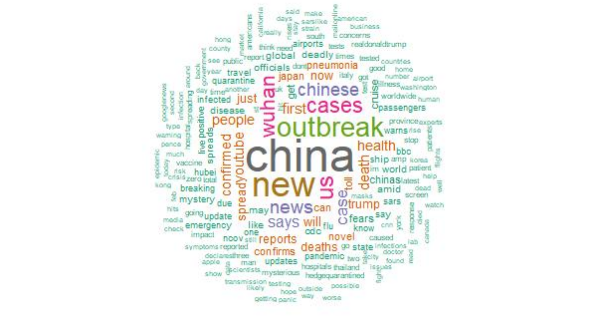
Word cloud showing the keywords appearing most frequently in tweets related to COVID-19.

This analysis used word clouds, which can provide a visual representation of text appearing in tweets. Word clouds highlight words according to frequency. In this study, the word clouds of frequently appearing words provided deeper insights into tweets related to COVID-19 posted by Twitter users. According to Figure 4, the most frequently appearing words were related to China, representing the first human cases of COVID-19 reported by officials in Wuhan City, China. Moreover, the word *new* shows the spread of a new virus, and the word *outbreak* also reflects the spread of a continuous epidemic. The secondary words in the word cloud are *Wuhan*, *death*, *health*, *people*, *spread*, and *confirmed*, depicting public perspectives regarding COVID-19.

**Figure 5:**
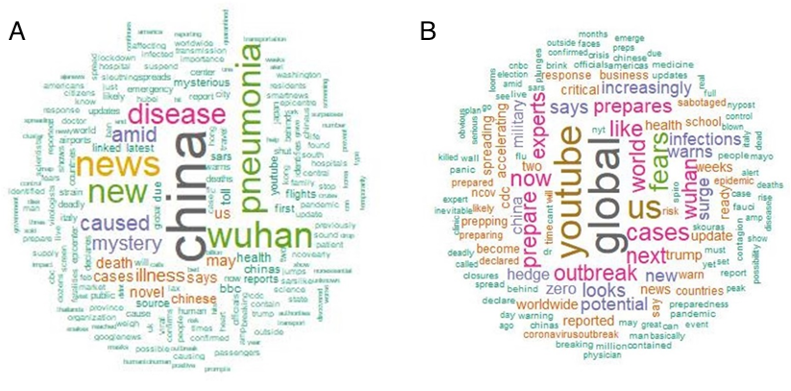
Word clouds of frequently mentioned keywords on Twitter related to the COVID-19 outbreak (A) and pandemic (B).

When considering the frequency of certain keywords in a specific search, we used the words *outbreak* and *pandemic* to examine different perspectives regarding different types of spread. Theoretically, an outbreak is a greater-than-anticipated increase in the number of endemic cases. An outbreak can be a single case in a new area. If an outbreak is not controlled, it can develop into an epidemic. From this perspective, the words with the highest frequency for *outbreak* were *China* and *Wuhan* (Figure 5A), which were the first country and city to report the outbreak. Other words relating to COVID-19 were *pneumonia*, which describes inflammation of the lungs in patients infected with COVID-19. Pneumonia was the pilot symptom mentioned in the outbreak period, when a number of patients with pneumonia were reported in Wuhan, China, leading the National Health Commission to declare a new epidemic. Officials attempted to find the cause and the first infected person, and they attempted to control the outbreak area. Other frequently appearing words include *disease*, *new*, *death*, and *mystery*, showing the point of view during the outbreak, that is, the period when COVID-19 had not reached worldwide levels; this analysis was restricted to the beginning of the COVID-19 outbreak, which was characterized by the mention of pneumonia in China.

A keyword frequency search was performed using the word *pandemic*, which is defined as a disease that affects a large number of people within a community, population, or region that spreads throughout multiple countries or continents. This meaning reflects the frequency of perception of COVID-19 in the context of a pandemic. The words most frequently mentioned with the word *pandemic* are *global*, *world*, *outbreak*, and *YouTube* (Figure 5B), which are During this time, the numbers of cases increased in locations worldwide. As a social media platform with billions of daily views, YouTube has tremendous potential to both support and hinder public health efforts regarding COVID-19 information. Social media was mentioned as the media of pandemic news trends. Moreover, other related words were *expert*, *prepare*, and *now*, showing an awakening toward the pandemic and the preparation and experts needed to stop it. The WHO declared COVID-19 a pandemic due to its worldwide spread, which was exacerbated by domestic travel. This is a normal occurrence after passengers arrive home from pandemic countries, leading to infection of family members or friends and spread of the disease to new, hard-to-control areas.

### Sentiment Analysis on COVID-19

**Figure 6:**
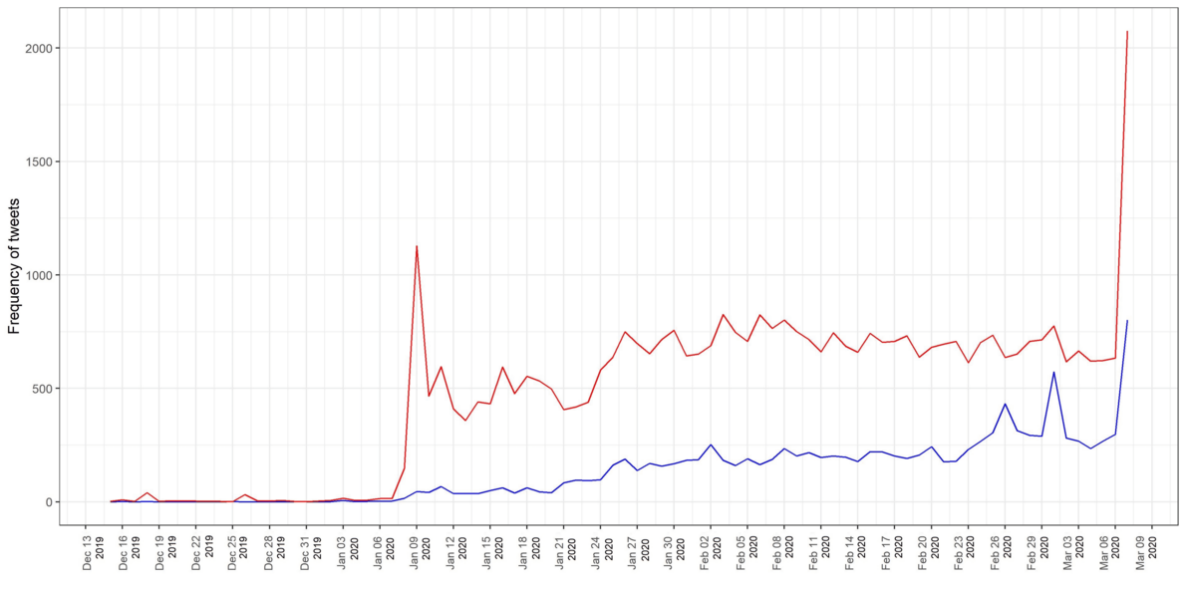
Sentiment analysis of negative (red line) and positive (blue line) tweets related to COVID-19.

Sentiment-level analysis further enriched the findings through the clear identification of negative and positive topics on COVID-19. The sentiment analysis found that 22.12% of the tweets (n= 23,887) contained positive sentiments, while 77.88% (n=84,103) contained negative sentiments. This signifies that Twitter users had a negative outlook toward COVID-19. According to Figure 6, between January 6 and 9, when the discovery of COVID-19 was officially announced, negative sentiment increased more than positive sentiment. During the period up to March 6, during which a high death rate and pandemic-level outbreak occurred, negative sentiment increased again when the number of persons affected by COVID-19 surpassed 100,000. COVID-19 continued to spread even as the WHO urged countries to increase their efforts to contain the spread, as mentioned earlier [29]. The public’s positive emotions increased as more information became available about prevention and protection of COVID-19, which is favorable for public health communication and promotion. The analysis showed expression of positive feelings through keywords such as *trust*, *protect*, and *safe*, and it demonstrated that the public still trusted experts and departments to help them get through the situation.

**Figure 7:**
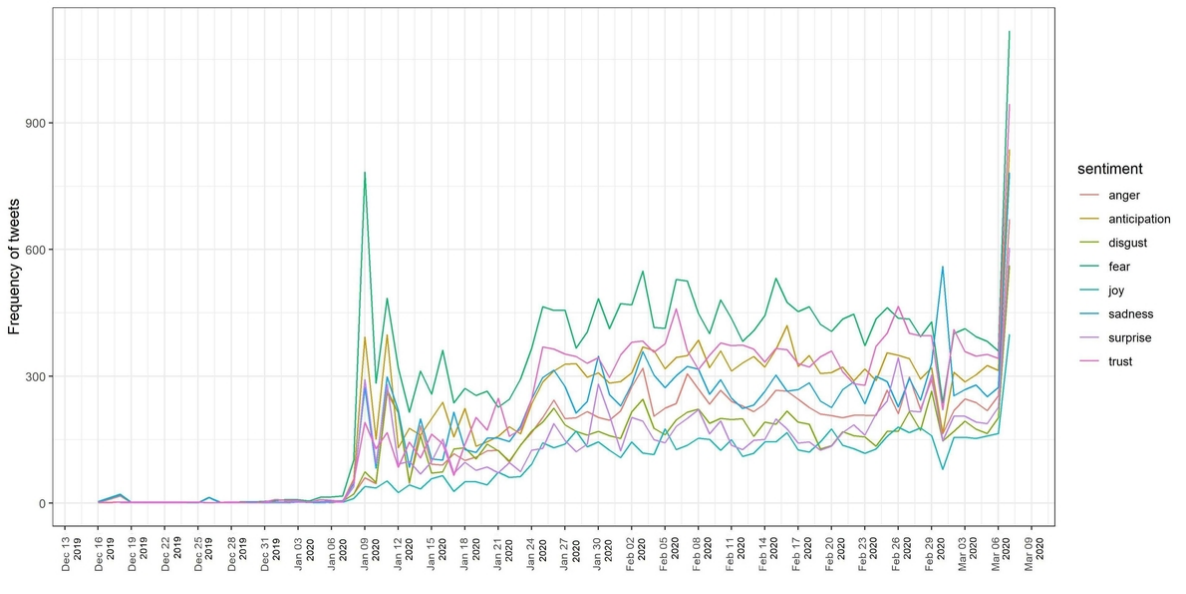
Sentiment analysis based on terms in the National Research Council sentiment lexicon.

**Figure 8:**
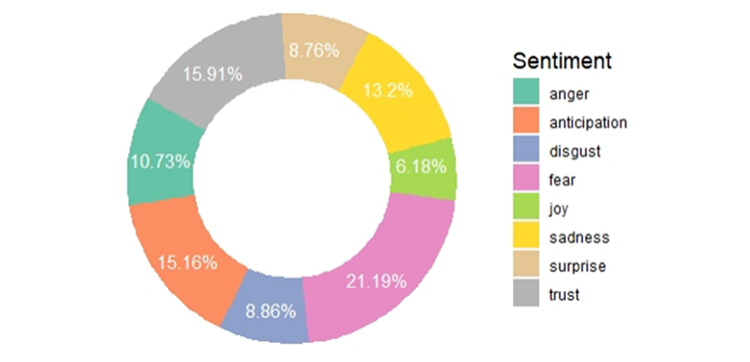
Sentiment wheel showing the emotional quotients of the studied tweets.

**Figure 9:**
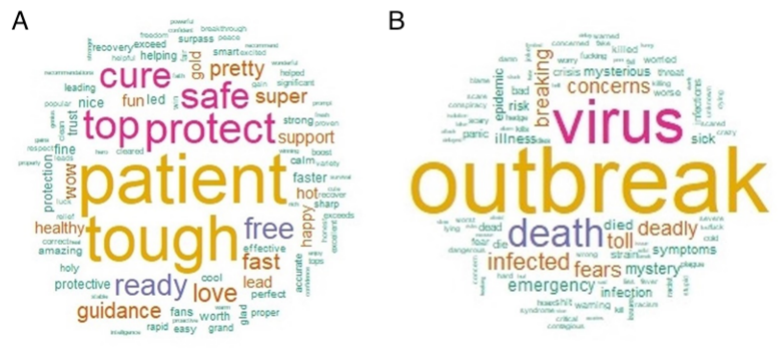
Word clouds of frequent positive (A) and negative (B) keywords related to the COVID-19 epidemic.

The analysis of the emotional quotient of the tweets using the NRC lexicon (Figure 7) found that over half of the tweets posted worldwide were defined by three emotions, namely fear, trust, and anticipation. Figure 8 shows that the tweets expressing the emotion of fear comprised approximately one fifth (21.19%, n=22,883) of the total tweets analyzed. Following the fear emotion was the trust emotion, which indicated that people were looking forward to recovery or to solutions from experts. Similarly, the emotion of anticipation was associated with almost 15.16% (n=16,371) of the tweets, which strengthens the positive sentiments of the people. Negative emotions, such as sadness, anger, and disgust, were seen in a portion of the tweets, with shares of 13.20% (n=14,254), 10.73% (n=11,588), and 8.86% (n=9,568), respectively. Only a small portion (6.18%, n=6,673) were categorized as joy, which is a positive emotion. These results show that people had a negative outlook toward COVID-19. As shown in Figure 9, the positive sentiment keywords in the tweets were *patient*, *protect*, *tough*, *safe*, and *cure*, while the keywords expressing negative sentiments were *outbreak*, *virus*, *death*, *infected*, and *fear*.

**Figure 10:**
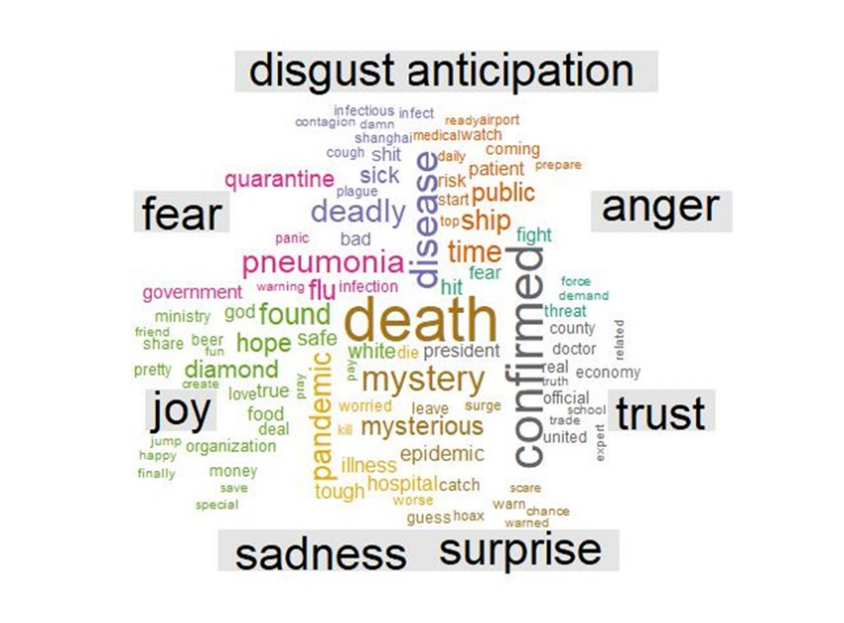
Word cloud showing the most frequently mentioned words and their related emotions (categorized by color).

For the next phase of the sentiment analysis, a word cloud was created using the most frequently used emotion words, which were categorized by color. The sentiments were part of the sentiment library in R (the textdata package), in which each emotion was separated with no overlap. The word cloud was developed based on the whole corpus of tweets. The results in Figure 10 show that the word *death* was tweeted most frequently in this context. As illustrated in Figure 10, words such as *death*, *mystery* (*mysterious*), *epidemic*, and *guess* were the most frequently used words related with the emotion of surprise. Words such as *pneumonia*, *flu*, *infection*, *panic*, and *quarantine* were tweeted frequently and were related with the emotion of fear. Words such as *disease*, *deadly*, and *sick* were tweeted frequently with the emotion of disgust. Words such as *pandemic*, *illness*, and *hospital* were frequently tweeted with the emotion of sadness. For the positive sentiments, people tweeted words such as *hope*, *safe*, and *diamond* to express the emotion of joy, while words such as *confirmed*, *doctor*, and *expert* were regularly used with the emotion of trust.

These results indicate that when most people thought about the COVID-19 pandemic during this period, they experienced negative emotions. Most of the users were surprised by the emergence of a mysterious disease with no prior information about how to treat it and the possibility of death. In addition, when the users talked about symptoms such as pneumonia, flu, or infection, they usually felt great fear.

### Topic Modeling

#### COVID-19–Related Topics and Themes

**Figure 11:**
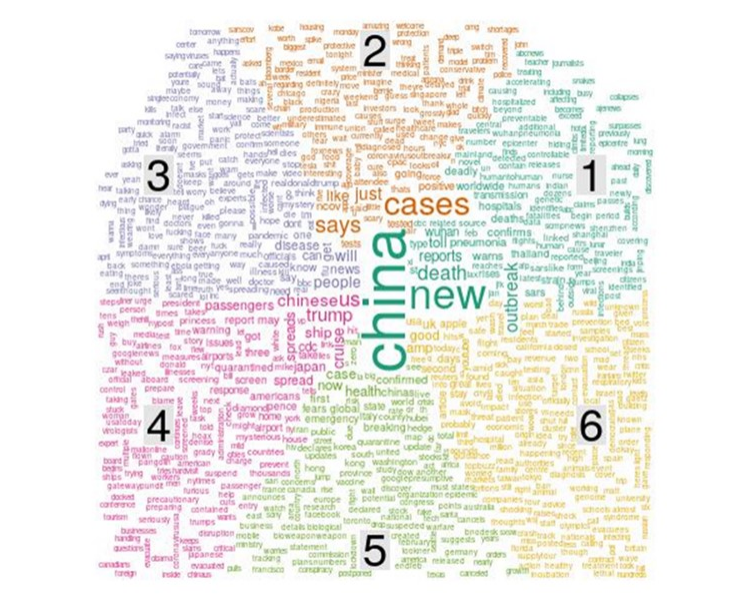
Word cloud showing the frequency of words associated with six identified topics: (1) reports on deadly pneumonia new cases and deaths of coronavirus outbreak from China; (2) the epidemic situation and confirmed cases of COVID-19; (3) knowledge about COVID-19 obtained from news reports; (4) the spread of COVID-19 from overseas to the US and how to control the disease; (5) health concerns and fear as COVID-19 is declared an emergency worldwide; (6) news and information reports on social media about the epidemic.

**Figure 12:**
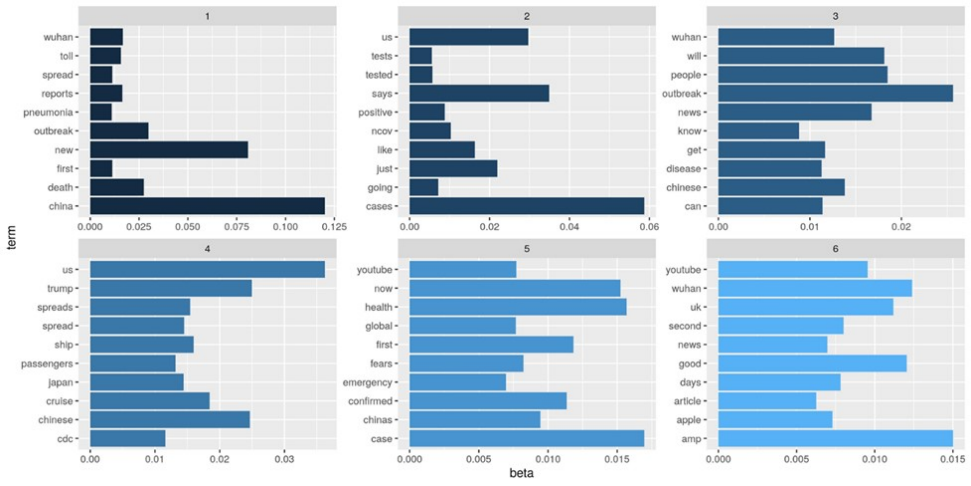
The per-topic-per-word probabilities produced by latent Dirichlet allocation by extracting the beta matrix.

In this section, the emergent topics and themes identified using topic modeling are summarized (Table 1). The objective of the topic modeling was to answer the research question: What are the emergent topical themes and discourses regarding COVID-19? We identified 6 topics based on the highest topic coherence. Figure 11 depicts a word cloud for the 6 topics, where the size of each word is proportional to the density p(word|topic). As shown in Figure 12, the top 10 most common words in each topic by beta value were considered in the study. This study used these words to provide each topic with a degree of semantic interpretation in the related contexts through relevant topic descriptions. The higher the beta value, the greater the possibility of a relatable word appearing in the category. By this approach, six topics were classified based on their per-topic-per-word probabilities (beta) as follows.

**Table 1:** The emergent topics and themes in tweets about COVID-19.

Topic	Ten most common words	Theme
Topic 1: Reports on new cases of deadly pneumonia and deaths from the COVID-19 outbreak in China	*China*, *death*, *first*, *new*, *outbreak*, *pneumonia*, *reports*, *spread*, *toll*, *Wuhan*	Theme 1: The emergency of the COVID-19 pandemic
Topic 2: The epidemic situation and confirmed cases of COVID-19	*Cases*, *going*, *just*, *like*, *nCov*, *positive*, *says*, *tested*, *tests*, US	Theme 2: How to control the COVID-19 pandemic
Topic 3: Public knowledge about COVID-19 from news reports	*Outbreak*, *people*, *will*, *news*, *Wuhan*, *Chinese*, *get*, *knows*, *disease*, *can*	Theme 3: Reports on the COVID-19 pandemic
Topic 4: The spread of COVID-19 from overseas to the US and how to control the disease	*US*, *Trump*, *Chinese*, *cruise*, *japan*, *passenger*, *spread*, *spreads*, *ship*, *CDC*	Theme 2: How to control the COVID-19 pandemic
Topic 5: Health concerns and fear as COVID-19 is declared an emergency worldwide	*Case*, *health*, *now*, *first*, *confirmed*, *China*, *fear*, *global*, *emergency*	Theme 1: The emergency of the COVID-19 pandemic
Topic 6: News and information reports on social media about the epidemic	*Amp*, *Wuhan*, *good*, *UK*, *YouTube*, *second*, *days*, *apple*, *news*, *article*	Theme 3: Reports on the COVID-19 pandemic

Topic 1 involves discussion related to the report of new cases of deadly pneumonia in China and the outbreak of COVID-19. Examples of keywords include *China*, *death*, *first*, *new*, *outbreak*, *pneumonia*, *reports*, *spread*, *toll*, and *Wuhan*. The word with the highest beta value is *China*. Topic 2 focuses on the epidemic situation and confirmed positive cases of COVID-19. Examples of keywords include *cases*, *going*, *just*, *like*, *nCov*, *positive*, *says*, *tested*, *tests*, and *US*. The words in Topic 3 collectively relate to what people learned from news reports about COVID-19 and the outbreak, with terms such as *outbreak*, *news*, *know*, and *disease*. The top words in Topic 4 describe the spread of COVID-19 and how to control the disease, while those in Topic 5 capture health concerns and fear regarding COVID-19 as an emergency. Some of the top 10 words in these topics are *wear*, *emergency*, and *health*, which are generally believed to be terms related to health concerns. Topic 6 is collectively associated with news and information reports on social media regarding COVID-19. The top terms in Topic 6 are *news*, *articles*, and *YouTube*.

The qualitative content analysis approach enabled the categorization of these topics into different distinct themes. As shown in Table 1, the sample tweets in each topic were categorized and identified according to six topics, which were then assigned to three different themes. The theme identified based on the keywords in Topic 1 and Topic 5 is “the emergency of the COVID-19 pandemic,” which provides information on the reporting of new cases and deaths during the COVID-19 outbreak and health concerns regarding the worldwide emergency. Sample tweets include “Top WHO official warned the world may be ‘dangerously unprepared’ for next pandemic as coronavirus outbreak spreads” and “The US health department declared the coronavirus a health emergency, 8 cases confirmed in the US, 259 dead over 11K infected in China.”

Topics 2 and 4 reflect the theme of “how to control the COVID-19 pandemic,” relating to the epidemic situation and confirmed cases of COVID-19 and its spread. Sample tweets include “Fox News’ Maria Bartiromo predicted ‘hundreds of thousands of US coronavirus cases: ‘I don’t want to panic anybody’” and “As the coronavirus grows and infects and kills more people, [US president] Trump slashed the budget for the CDC [US Centers for Disease Control and Prevention] that controls disease.” Topics 3 and 6 reflect the final theme of “reports on the COVID-19 pandemic,” which is about the channels receiving news and information about COVID-19; example tweets include “#Coronavirus has been dominating the news, but how much do we need to worry about it” and “China spent the crucial first days of the Wuhan coronavirus outbreak arresting people who posted.”

#### COVID-19 Outbreak–Related Themes

**Figure 13:**
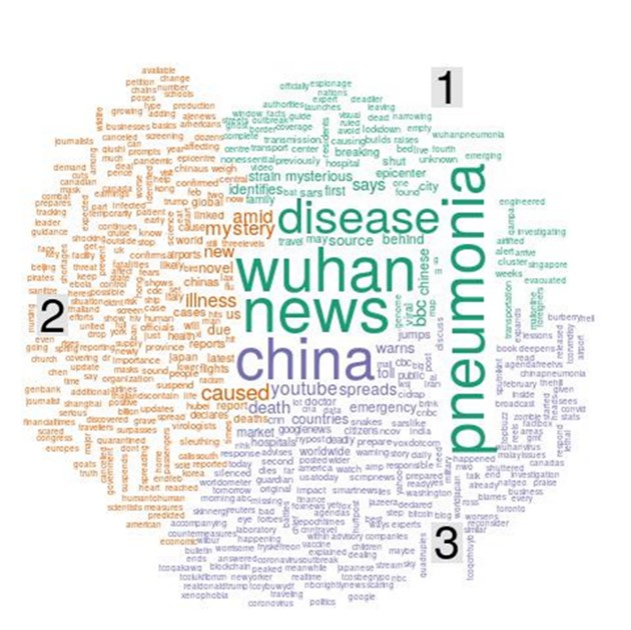
Word cloud and topic modeling of keywords related to the COVID-19 outbreak, organized into three topics: (1) The new strain of pneumonia identified in Wuhan, China; (2) the mysterious illness caused by the novel virus; (3) the warning from China that the death toll of COVID-19 could increase.

To explore the keywords for themes reflecting topics related to the COVID-19 outbreak, we used word clouds and topic modeling to generate themes and determine the co-occurrence of topic keywords related specifically to the *outbreak* of COVID-19. The results are shown in Figure 13. The main three issues of public concern regarding the COVID-19 outbreak were the COVID-19 illness, the status of the outbreak in Wuhan, China, and the situation in the news. The high-frequency keywords regarding these issues can be divided into three topics: the new strain of pneumonia identified in Wuhan, China; the mysterious illness caused by the novel virus; and the warning from China that the death toll of COVID-19 could increase.

As shown in Table 2, Topic 1 captures discussions regarding the new strain of pneumonia identified in Wuhan. Examples of keywords include *news*, *Wuhan*, *pneumonia*, and *disease*. Topic 2 involves mentions of the new mysterious illness caused by a virus. Examples of keywords include *new*, *cause*, and *mystery*. Topic 3 includes mentions of the death toll in China. Examples of keywords in this topic include *China*, *death*, and *emergency*. The theme of Topic 1 and Topic 2 concerns the new mysterious illness and new strain of pneumonia caused by a virus identified in Wuhan. Based on Topic 3, another theme was identified, namely the warning by China that the death toll could jump.

**Table 2:** The emergent topics and themes related to the outbreak of COVID-19.

Theme and topics		Related words	
**Theme 1: Mysterious new illness**			
	Topic 1: New strain of pneumonia identified in Wuhan		*Wuhan*, *pneumonia*, *identifies*, *strain*
	Topic 2: Mysterious new illness caused by virus		*Novel*, *mystery*, *caused*, *illness*
**Theme 2: China warns that death toll may jump**			
	Topic 3: China warns that the death toll could jump		*China*, *spreads*, *jumps*, *warns*, *toll*

## Discussion

### Principal Findings

This Twitter data analysis can be used to explain the public awareness and perception of the COVID-19 pandemic. Based on public awareness, the data were divided into three main stages in relation to the timeline of the epidemic. The early or incubation stage (Stage 1) was the phase in which the severity and the spread of COVID-19 began to increase. The public started to become aware of the severity and rapid spread of the disease and then became afraid, especially when the WHO announced the emergence of a novel and mysterious virus related to pneumonia. This result is in accordance with a previous study [31], which explained the different stages of the public’s attention to COVID-19. It is necessary to avoid undermining the possibility of a serious outbreak during the incubation stage [32]. Stage 2 was the worldwide epidemic stage. In Stage 3, the public began to become more aware, as scientific and medical understanding of the disease increased and governments announced the need for social distancing and lockdowns. This was a stable stage from the perspective of public perception, where public awareness tended to be positive.

The mentions of COVID-19 symptoms demonstrated that fever was recognized as the major symptom of COVID-19, which is in accordance with research results stating that fever is seen in 94.3% of cases and is the most common symptom present at the onset of illness (87.1%), followed by coughing (36.5%) and fatigue (15.7%) [33,34]. These common symptoms, including fever and cough, remain consistent across several studies [35]. Fever is understood to be a precursory indicator of COVID-19. After that, the virus progresses to the respiratory system, causing pneumonia and a severe cough [27]. Coughing is a significant symptom in the late stage of fever. However, other symptoms are mentioned, such as a stuffed nose and headache.

The results of the sentiment analysis of tweets related to COVID-19 showed that the most important keyword was *outbreak*, which was related to the starting point of the disease in Wuhan, China. The keywords related to public awareness were different according to the stage of the spread; this includes public emotion, which was mostly more negative than positive, where *fear* was the most negative word [36,37]. In previous disease pandemics, negative sentiments were generally prevalent in social media [38]. A study by Raamkumar [39] showed that *fear* was the most negative sentiment expressed about COVID-19. As the epidemic progressed, however, public sentiment tended to be more positive because additional news was being reported at this stage. The result is in accordance with previous research showing that on social media, people’s interests are related to the latest news and major events regarding infectious diseases [40]. Studies have also indicated that people pay attention to and search for disease-related words as the spread of infectious disease changes [41].

The COVID-19 crisis has stimulated great public concern worldwide. Users on Twitter discussed six main topics across three main themes of (1) the emergency of the COVID-19 pandemic, (2) how to control the COVID-19 pandemic, and (3) reports on the COVID-19 pandemic. Previous studies have also shown that prevention and control procedures, including quarantine, as well as reports on confirmed cases and medical treatments were major themes during previous disease outbreaks [42-45].

### Practical Implications

Policy makers should recognize that Twitter data can be used to explore levels of public awareness and emotions about the COVID-19 pandemic. It is important to note that the levels of public awareness are dynamic, which can be observed from the two or three awareness peaks in a period of just a few months in this study. The results also suggest that people express negative emotions and share both information and misinformation via social media platforms during the different stages of the disease. People usually feel great fear during pandemics. The government should synchronize the flow of information and combat “fake news” about the pandemic to diminish this fear. It is also suggested that the government should mitigate the impact of this emotion by implementing countermeasures and building national surveillance systems to examine web-based content, including social media, to better understand the emotions of the public. Misinformation on the internet can create mass panic and result in negative actions. There is a need for a more proactive public health presence on social media. In addition, governments should clearly convey and communicate information regarding COVID-19 to their populations. Key decisions and actions must be informed by accurate and timely data on the delivery and uses of health services throughout all phases of the COVID-19 pandemic.

As COVID-19 continues to spread, more efforts from governments and public health agencies are needed to answer ongoing questions. Twitter users focus on discussing and reacting to health concerns, public health interventions, and controlling the pandemic. This type of information helps governments to understand which public health messages are resonating. For example, how can governments respond in the short term as well as the long term to keep people safe? Governments at all levels need to improve their responses. Governments and public health entities need to ensure that health care systems are prepared to handle increasing numbers of cases. Community-based health care is an essential part of primary care to respond to the COVID-19 pandemic. The recognition of public concern and awareness can help governments understand what the public is thinking about the disease at a particular time. When the results are connected, a valuable health care resource can be established to develop a future plan.

### Limitations

This study contains several limitations. First, it is worth mentioning that this study used keywords related to COVID-19 to investigate trends and frequencies of keywords. The list of selected keywords may have been incomplete. The keywords used in this study can be extended to cover the search of tweets by combining keywords related to COVID-19 and its symptoms. Further research could aim to identify the most relevant set of keywords with a high level of detail based on the number of tweets that include symptoms and other keywords. Second, this research was performed in the early phase of the pandemic, which ultimately spread worldwide. This limited the scope of public awareness of the total picture of the pandemic as well as the pandemic cycle. Thus, a study focusing on public concern after the mentioned period may provide useful results for comparison. Third, although LDA was advantageous in extracting hidden themes, the scientific quality of the themes should be further validated. Furthermore, researchers can play a greater role in extracting themes. Moreover, it is necessary to reduce bias, which may occur when identifying topic themes using topic modeling. Finally, it may be difficult to identify perfect sources of information on social media because the amount of information regarding COVID-19 is overwhelming. This research collected data from Twitter only; further research should use other resources such as mass media or other data sources in addition to social media information.

## Abbreviations

API: application programming interface
LDA: latent Dirichlet allocation
NLP: natural language processing
NRC: National Research Council
TF-IDF: term frequency-inverse document frequency
WHO: World Health Organization
